# Gender difference in pre-clinical liver-directed gene therapy with lentiviral vectors

**DOI:** 10.3389/ebm.2025.10422

**Published:** 2025-04-25

**Authors:** Efrain Guzman, Cheen Khoo, Deirdre O’Connor, Gayathri Devarajan, Sharifah Iqball, Bernard Souberbielle, Kyriacos Mitrophanous, Yatish Lad

**Affiliations:** Oxford Biomedica (UK) Limited, Oxford, United Kingdom

**Keywords:** lentiviral vectors, adeno-associated virus, AAV, liver, PAH, gender, murine

## Abstract

Viral vector-based therapies are effective therapeutics for the correction of several disorders, both in mouse models and in humans. Several pre-clinical studies have demonstrated differences in transduction efficiencies and therapeutic effect between male and female mice dosed with AAV-based gene therapy product candidates. Here, we report gender-specific transduction and transgene expression differences in mice dosed systemically with lentiviral vectors (LVV). Male mice systemically dosed with LVV carrying the reporter gene luciferase showed at least a 12-fold higher expression of luciferase and a higher vector copy number (VCN) in their livers compared with female mice. Lastly, PAH^Enu2^ male mice dosed with a LVV carrying the human phenylalanine hydroxylase (PAH) transgene were observed to have a higher VCN than their female littermates. These findings suggest that sex-based differences initially observed in AAV-mediated therapies also apply to LVV, but the exact mechanism remains to be determined.

## Impact statement

There are many reports of lower expression of adeno-associated virus (AAV) transgenes in female compared to male mice. The reason for this difference has not been explained with different theories proposed. This report is the first report that describes the same phenomenon with a different viral vector, i.e. lentiviral vectors, and suggests universality for the phenomenon, not exclusive to AAV, thus excluding a receptor-specific phenomenon explanation. It is hoped that this report will stimulate other groups working with lentiviral vectors to confirm, or refute, this observation from their own data, and potentially other groups working with viral vectors other than lentiviral vectors and AAV. If confirmed, this report will help understand the reasons for this sex-difference phenomenon in murine models and stimulate specific experimental work to explain the phenomenon. Whether this phenomenon is also observed in humans, this is not currently known, but it should also be investigated in humans.

## Introduction

Lentiviral vectors (LVV) are an established platform for gene delivery. LVV integrate into the host’s genome providing long-term expression of the therapeutic transgene. They are the preferred platform for the *ex vivo* transduction of haematopoietic cells or generation of CAR-T cells in clinical applications with good safety and efficacy profiles with several approved products on the market [1]. There are numerous pre-clinical reports of *in vivo* delivery of LVV targeting the liver for the correction of metabolic disorders [2–8], however none have yet to be translated into the clinic in contrast to AAV based vectors.

Achieving optimal transduction and therapeutic transgene expression following *in vivo* delivery is essential for demonstrating the efficacy of gene therapy candidates during pre-clinical studies. In general, laboratory mice are the chosen model to do so for gene therapy products. However, there are multiple reports of reduced transduction efficiency, lower expression of the transgene and product efficacy in female mice compared to male mice in liver-directed gene therapy studies with adeno-associated virus (AAV) [9–12].

Several mechanisms for the observed gender differences in transduction and transgene expression in mice have been proposed [10]. It is unlikely that the observed differences are solely due to differential expression of the AAV binding receptor(s) on liver cells, as gender differences have been demonstrated in mice across different AAV serotypes that bind and enter through unrelated receptors [10]. In addition, whether the transduction efficiencies and transgene expression differences in the livers of male vs. female laboratory mice is strain-specific or serotype-specific is not currently known. It has been suggested that transgene expression difference observed in both genders of mice could be due to specific binding of the AAV to liver proteins in an androgen-dependent pathway [10]. Whether this hypothesis applies solely to AAV based vector or to other vector formats remains to be tested.

With AAV based vectors, several routes of delivery have been evaluated for liver-based gene therapies targeting the liver in both pre-clinical models and in the clinic. Intravenous (IV) administration is by far the simplest route of administration that can be translated from pre-clinical models to patients. In addition to IV administration, portal vein (PV) and hepatic artery injections have also been evaluated, as well as direct administration into the liver tissue.

In this study we evaluated *in vivo* transduction efficiency of hepatocytes and transgene expression of the liver with LVV in both male and female mice. To our knowledge, such a comparison of transduction and transgene expression of vector-encoded transgenes has not been reported with LVV. Here, we report an increase in the transduction efficiencies and transgene expression in the livers of male laboratory mice compared to female mice following delivery of LVV.

## Materials and methods

### Cell lines

The human embryonic kidney cell line expressing the SV40 large T antigen (HEK293T) was obtained from ATCC (LGC Standards, Teddington, United Kingdom) and cultured in Dulbecco’s modified Eagle’s medium (DMEM, Merck Life Science, Dorset, United Kingdom) supplemented with 10% heat-inactivated fetal bovine serum (FBS; ThermoFisher Scientific, United Kingdom), 2 mM L-glutamine (Merck Life Science) and 1% non-essential amino acids (NEAAs; Merck Life Science). HEK293T cells adapted to culture in suspension phase were maintained in Freestyle™ 293 Expression Medium (FS; ThermoFisher Scientific) with 0.1% cholesterol lipid concentrate 250X (CLC; ThermoFisher Scientific) (FS + 0.1% CLC).

### Vector construction

Third generation lentiviral vector carrying Gaussia luciferase cDNA (LVV-GLuc) was generated as follows: the coding sequence for GLuc followed by a T2A and the enhanced green fluorescent protein (eGFP) sequences were synthesized by ThermoFisher Scientific and cloned into a minimal self-inactivating, third generation HIV-1 transfer vector downstream of a mouse transthyretin promoter preceded by a synthetic enhancer [13], also referred to as ETpro. OXB-401 was generated by cloning synthetic cDNA encoding the human codon optimized phenylalanine hydroxylase gene (PAH, ThermoFisher Scientific) into a minimal self-inactivating, third generation HIV transfer vector downstream of the ETpro mentioned above. OXB-Null did not contain any coding sequences downstream of the ETpro.

### Vector production

Third generation, VSVG-pseudotyped LVV were produced and titrated in HEK293T cells as described before [14] and resuspended in a formulation buffer of Tromethamine, NaCl, Sucrose and Mannitol (TSSM) [15] which was also used as vehicle control.

### Mice

All animal studies were carried out by Charles River (CR) Discovery Services and approved by the local ethics committee. For the initial gene transfer study, 5–8 week-old male and female BTBR mice (BTBR T^+^ Itpr3/J, n = 5/group, The Jackson Laboratory) of an average weight of 25g, were dosed with 1.5 × 10^10^ TU/kg [intravenously (IV) via the tail vein] or 7.5 × 10^9^ TU/kg [via the intrahepatic portal vein (PV)] of LVV-GLuc (n = 5/group) or with TSSM buffer (n = 1/group) either via IV or PV routes (Table 1). Dosing was performed according to the average weight of the male or female mice respectively. Due to the maximum volume that could be administered by intrahepatic portal vein injection limited to 100 µL compared to the 200 µL by tail vein administration there is a 2-fold lower total dose administered to the mice via this route. The study designs are summarized in Table 2.

**TABLE 1:** Study design for the long-term gene transfer study.

Name	Sex	Route	Treatment	Vector dose (TU/kg)
Group 1	Female	IV	TSSM	N/A
Group 2	Female	IV	LVV-GLuc	1.5 × 10^10^
Group 3	Male	IV	TSSM	N/A
Group 4	Male	IV	LVV-GLuc	1.5 × 10^10^
Group 5	Female	PV	TSSM	N/A
Group 6	Female	PV	LVV-GLuc	7.5 × 10^9^
Group 7	Male	PV	TSSM	N/A
Group 8	Male	PV	LVV-GLuc	7.5 × 10^9^

**TABLE 2:** Study design for the OXB-401 study.

Name	Sex	Route	Treatment	Vector dose (TU/kg)
Group 1	Female	IV	OXB-Null	4 × 10^10^ TU/kg
Group 2	Male	IV	OXB-401	4 × 10^10^ TU/kg
Group 3	Female	IV	OXB-Null	4 × 10^10^ TU/kg
Group 4	Male	IV	OXB-401	4 × 10^10^ TU/kg

Whole body bioluminescent imaging was performed as described before [9, 16] on days 1, 8, 15, 22, 29, and then once every 2 weeks until day 83 post dosing. Two mice each from Groups 2, 4, 6, and 8 were sacrificed at day 29 to determine VCN. D-luciferin was administered (150 mg/kg) intraperitoneally (IP) and 10 min post substrate injection, dorsal and ventral images were taken, and the scaled images quantified.

For the second study, BTBR-*Pah* ^enu2^/J mice [17], also referred to as Pah^enu2^) were obtained from The Jackson Laboratory and dosed IV with TSSM buffer (200 µL), OXB-Null (4 × 10^10^ TU/kg) or OXB-401 (4 × 10^10^ TU/kg) and observed for the duration of the study (85 days). All animals were euthanized at the end of the studies and the livers collected and snap-frozen.

### Genomic DNA extraction from liver tissues

Liver tissues from median and right lobes were weighed and 22 mg to 26 mg were homogenised in PBS using the TaKaRa^®^ BioMasher standard micro homogenizers. Genomic DNA was extracted using DNeasy Blood and Tissue Kit (Qiagen) following the manufacturer’s instructions.

### Quantification of integrated vector copies (VCN)

Duplex qPCR (QuantStudio 7, Thermo ABI) was performed on the liver-extracted DNA to obtain LVV integrated VCN per cell as described before [7] using the transferrin receptor protein 1 (*tfrc*) as a housekeeping gene. The primer probe set for *tfrc* was purchased from (ThermoFisher catalogue number 4458366) and primer probe set for HIV Ψ were synthesized by ThermoFisher.

The formula to calculate integrated VCN is as follows:

$$\text{VCN}=\frac{\text{Copies}\;\text{of}\;\text{Psi}\;}{\text{Copies}\;\text{of}\;\text{tfrc}\;\left(\text{divide}\;\text{by}\;2\right)}$$

### Statistical analysis

Data analysis and generation of graphs was performed using GraphPad Prism v.9 for Windows (GraphPad, San Diego, CA, United States) with descriptive statistics. Normality tests were used to evaluate the Gaussian distribution of data. Normally distributed data were analyzed using ordinary one-way ANOVA, and data not normally distributed were analyzed using Kruskal-Wallis and Dunn’s multiple-comparisons tests.

## Results

### Long-term expression of LVV-encoded transgenes in mice treated systemically

**FIGURE 1:**
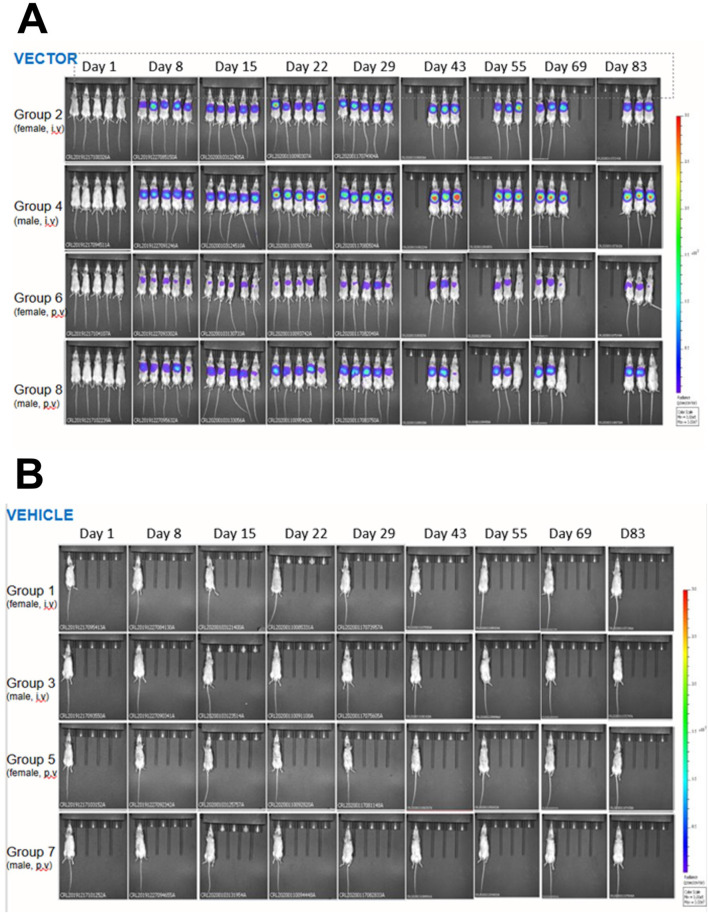
*In vivo* bioluminescent imaging of the expression of LVV-encoded luciferase in mice dosed with LVV- GLuc **(A)** or the vehicle TSSM **(B)** via either the intravenous (IV) or the portal vein (PV) routes. Mice were imaged as described in *Materials and Methods*.

An initial *in vivo* gene transfer study was designed to determine the transduction efficiency of LVV carrying the luciferase transgene (Table 1). Mice were dosed with pre-clinical grade preparations of LVV-GLuc, delivered via the intravenous (IV) or portal vein (PV) routes. *In vivo* imaging was carried out at regular intervals to determine transgene expression over time. LVV-encoded luciferase activity was observed as soon as day 8 and until the end of the study on day 83 (Figure 1A). Two mice, from Groups 2, 4, 6, and 8, were sacrificed at day 29 to determine VCN with the remaining three mice continuing to day 83. Luciferase expression, measured by whole body luminescence, was observed almost exclusively within the anatomical area which corresponds to the liver and was observed across all vector treated groups, males and females, and via both routes of delivery, IV and PV, from day 8 to the end of the study at day 83 (Figure 1A). In contrast, no luciferase expression was observed in the TSSM-treated groups (Figure 1B).

**FIGURE 2:**
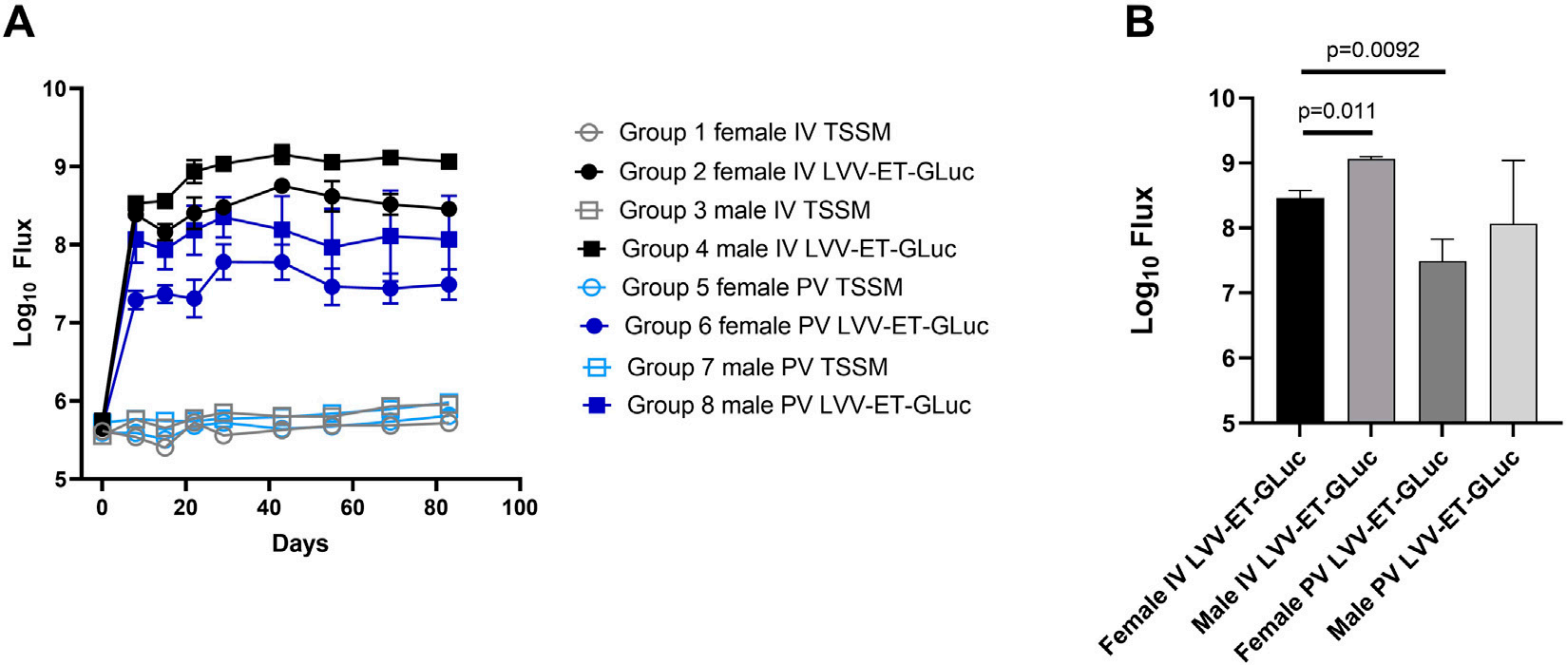
Quantification of luciferase expression of LVV-encoded luciferase in mice dosed with LVV-GLuc. **(A)** Quantification of *in vivo* expressed luciferase of all groups over time. **(B)** Quantification and statistical analysis of the *in vivo* expressed luciferase of all groups at day 83.

A quantitative analysis of luciferase expression *in vivo* showed that the maximum transgene expression was achieved between days 22 and 43 post-dosing and was sustained until the end of the study at day 83 (Figure 2A). Luciferase activity in the TSSM-treated group was considered background activity. *In vivo* expression of luciferase was higher in the groups dosed IV compared to the groups dosed PV at all time points (Figure 2A). It should be noted that the IV groups had a 2-fold higher dose of vector than the PV groups. A final statistical analysis at the end of the study showed that male mice dosed with LVV-GLuc had higher expression of luciferase than females dosed with the same vector in both IV (12-fold, p = 0.011) and PV (12-fold, p = 0.3867) routes (Figure 2B). In comparing routes of delivery within female and male groups, IV delivery of the vector resulted in higher luciferase expression than PV administration in both females (13-fold, p = 0.0092) and males (13-fold, p = 0.1498).

In conclusion, this analysis of the LVV *in vivo* gene transfer study demonstrated stable transgene expression out to the end of the study at day 83 and that as observed with AAV based vectors there is a higher transgene expression in the male mice compared to the female mice. In addition, the IV route mediated an increase in transgene expression compared to the PV route greater than the two-fold increase in vector dose administered via this route.

### Transduction efficiency differences between male and female mice

To determine if the differences in transgene expression observed above were due to differential transduction efficiencies *in vivo*, integrated vector copy number (VCN) analysis was carried out on the livers of all animals at the end of the study. Two mice from each group were euthanised at day 29 and VCN/cell was measured in the median and right liver lobes. VCN/cell was 4.8-fold higher in males (0.24 VCN/cell) compared to females (0.05 VCN/cell) dosed through the PV route (p = 0.0218) but with mice dosed through the IV route there was no significant difference between the male (0.24 VCN/cell) and female mice (0.21 VCN/cell, p = 0.3935, result not shown).

**FIGURE 3:**
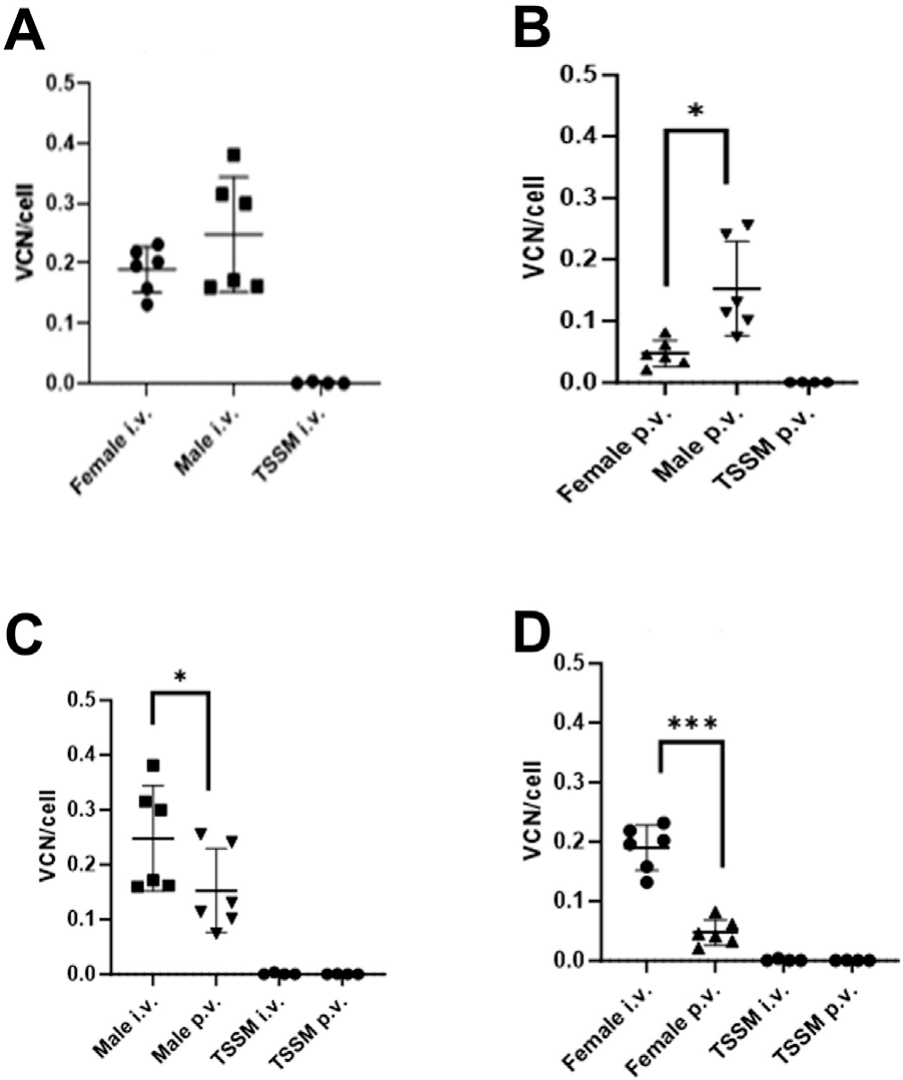
Integrated vector copy number (VCN) in livers of male and female mice dosed via two different routes of administration. Livers were collected at day 83 post dosing. **(A)** Male vs. female dosed IV. **(B)** Male vs. female dosed PV; * indicates p = 0.014. **(C)** Males dosed IV vs. PV; * indicates p = 0.0008. **(D)** Females dosed IV vs. PV; *** indicates p = 0.0301.

In the remaining mice taken to the end of the study at day 83, VCN was higher overall in males compared to females for both IV and the PV routes (Figures 3A, B). VCN obtained from male mice in the IV group was 0.25 VCN/cell and not significantly different to VCN obtained from female mice (0.20 VCN/cell, p = 0.4237, Figure 3A) and similar to that observed in the mice taken at day 29. With the mice administered vector via the PV route there is a 5.9-fold increase in VCN/cell in male (0.176 VCN/cell) versus female mice (0.03 VCN/cell, p = 0.014, Figure 3B), which is consistent with the significant difference seen at day 29. In comparing routes of delivery within sexes, male mice dosed IV had 2-fold higher VCN/cell compared with male mice dosed via the PV route (p = 0.0301, Figure 3C). Similarly, female mice dosed IV had 4.9-fold higher VCN/cell compared with female mice dosed via the PV route (p = 0.0008, Figure 3D).

In summary there was a 12 to 13-fold difference in gene expression (from Figure 2 data), a higher VCN in male vs. female mice, and a higher VCN in the IV-dosed vs. PV-dosed groups, therefore we can conclude that the difference in transgene expression between these groups can be partly attributed to differences in transduction efficiencies.

### Differences in *in vivo* transduction efficiencies between male and female mice using a clinically-relevant therapeutic vector and transgene

**FIGURE 4:**
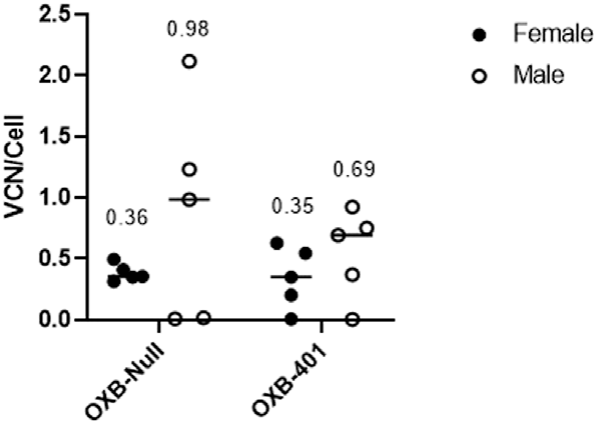
Differential transduction efficiencies in male vs. female Pah^enu2^ mice dosed IV with either OXB-401 or OXB-Null. Median VCN is shown for each group at day 85 post dosing. No significant differences were observed between the groups (p > 0.05).

To determine if the differences in transgene expression observed above were only seen using the reporter gene Gaussia luciferase, an analysis was conducted on a *in vivo* study using a therapeutic cargo and a mouse model of a human disease. OXB-401 is a third-generation LVV carrying a full-length copy of the human codon-optimized phenylalanine hydroxylase (PAH) open reading frame driven by the liver-specific ET promoter (ETpro). Both male and female Pah^enu2^ mice (n = 5) were dosed intravenously with OXB-401 or OXB-Null as described in *Materials and Methods*. VCN analysis was performed on the livers harvested at the end of the study at day 85 days post-dosing. Male mice showed 2 to 3-fold higher but not significant (p > 0.05) VCN/cell than females in both treatment groups (Figure 4).

When the VCN/cell data in this study were compared with the data from the previous experiment it was observed that male mice had an average of 0.54 VCN/cell and females had an average of 0.25 VCN/cell, however these differences were not statistically significant (p = 0.1792). The spread of the data suggested that the transgene itself (GLuc, PAH or Null) does not have a significant effect on transduction efficiencies (data not shown).

These results are further evidence of the differential LVV transduction efficiencies in laboratory mice.

## Discussion

In this report we have shown that the liver expression of a reporter gene administered *in vivo* using a systemic lentiviral vector was significantly higher in male mice compared to female mice. Vector copy number per cell was also higher in male mice compared to female mice. In addition, an increased trend in transduction between male and female, although not significant, was also observed in a therapeutic model of disease with both the therapeutic vector, OXB-401 and a control vector OXB-Null.

To date there have been no reports comparing the expression of *in vivo*-delivered LVV between male and female mice. Our initial LVV *in vivo* gene transfer studies were designed to identify the optimal route of delivery for the transduction of the liver, to assess the safety profile of pre-clinical grade vector batches and to determine duration of the reporter transgene. The current study was designed using both male and female outbred laboratory mice. The luciferase signal was observed within 8 days and peaked between 22 and 43 days post dosing and was stable until the end of the study at day 83 post dosing, indicating long term expression of the transgene following a single administration of the vector. We and others have demonstrated stable transgene expression in other tissues and the liver in pre-clinical models [18]. In the clinic, with a gene therapy for wet-AMD, we have demonstrated stable and long-term transgene expression from a lentiviral vector in the eye for greater than 6 years [19].

In our studies, IV delivery of LVV post 83 days resulted in a 12-13-fold increase in luciferase activity than PV delivery during *in vivo* imaging in both male and female mice. While there was a two-fold increase in vector dose administered IV compared to the PV route due to constraints in total volumes able to be administered this only partly provides a reason for the increase in luciferase signal. Integrated vector copy number (VCN) analysis, carried out at the end of the study, demonstrated that IV dosing resulted in higher (2 to 5-fold) VCN compared to PV dosing (Figures 3A–D). With AAV based vectors, hepatic artery delivery has been assessed in pre-clinical models [20] and unlike our study they found that intravenous injection of AAV2 resulted in 4-10 fold less hepatic transduction when compared to intra portal injection [21]. For humans, portal vein and hepatic artery injections are more invasive than peripheral intravenous injection and need trained individuals who are experts in interventional radiology or surgical procedures, therefore increasing the complexity of the therapeutic approach compared to an approach based on peripheral injection.

When we compared the transgene expression differences between genders, we observed a higher luciferase expression in mice dosed via the IV route and the PV route post 83 days transduction. The increase was observed as soon as 8 days post dosing in both the IV and PV groups. A similar finding has also been reported with AAV-Luciferase dosed mice [16] This difference in transgene expression has been extensively reported with AAV based vectors in laboratory mice and this is the first report of this observation with LVV.

When VCN/cell analysis was performed at day 29 and day 83 there is a trend of an increase in transduction efficiency in male mice compared to female mice. These increases in VCN/cell may partly account for the increased expression in male versus female mice but other contributing factors may also be involved. To our knowledge this is also the first report of sex-specific differences in LVV transduction efficiencies and transgene expression.

To determine if the differences observed in VCN/cell using the reporter gene luciferase and outbred laboratory mice were specific to this study, a study was conducted using a therapeutic transgene and a mouse model of a human disease. OXB-401 is a third-generation LVV carrying a full-length copy of the human codon-optimized phenylalanine hydroxylase (PAH) gene driven by the liver-specific ET promoter (ETpro). Pah^enu2^ (BTBR-*Pah* ^*enu2*^/J) mice carry a T835C missense mutation, yielding an F263S single amino acid substitution that causes severely diminished PAH catalytic activity in homozygotes and provide a model of severe phenylketonuria. VCN analysis from the livers of Pah^enu2^ mice harvested 85 days post-dosing demonstrated a higher, but not statistically significant, change in transduction efficiency between male and female mice (Figure 4) with both therapeutic OXB-401 and the OXB-null vectors. Interestingly, two previous publications also reported sex-specific differences in Pah^enu2^ mice systemically dosed with AAV carrying a human PAH cDNA transgene [11, 22]. These results are further evidence of sex-differential LVV transduction efficiencies in at least two strains of laboratory mice. In our studies we used the liver-specific promoter ETpro, and so it would be important to determine if other promoters result in same or different results.

It has been suggested that androgens are likely responsible for the enhanced AAV transduction in male mice as castration was shown to reduce AAV transgene expression, whereas treatment of female mice with testosterone improved AAV transduction to levels observed in males [10, 23]. Additionally, AAV transduction differences between male and female mice have also been reported in organs other than the liver [24]. Our data indicate that sex-specific differences in AAV transduction efficiencies also apply to VSVG-pseudotyped LVV, but further analysis is required to better understand the mechanism of the gender differences observed in this report, including the effect of LVV envelopes other than VSVG. To date, these gender-specific differences have not been reported in species other than laboratory mice, as most AAV and LVV-related pre-clinical studies only use mice as the model species. When the gender difference expression is also confirmed by other groups, it would be important to assess the relative effects of different LVV envelops. In addition, the effect of age and sexual maturity has not been evaluated in pre-clinical studies. To evaluate gender-specific differences in non-human primates or even humans, the design of relevant studies must be carefully evaluated to obtain statistically significant values. Whether these effects are also relevant in other viral and non-viral vectors, the exact mechanism and whether this also applies to other species, including humans, remains to be investigated. In future studies, it would be of interest to evaluate the efficacy of transduction and transgene expression in *in vitro*-transduced cells, for example freshly isolated hepatocytes, from male and female subjects. Indeed, if the gender effect is caused by some hormonal factors, the gender difference would not be seen *in vitro* between female and male cells. Whether there is gender difference on cell lines originally isolated from female or male donors (from either mice of humans) would be a complementary and additional way to tackle this question. On the same line, the inverse *in vitro* experiments were conducted by Davidoff and colleagues who showed that androgens increased AAV-transgene expression *in vitro* by upregulating its receptors [10].

With the increase in transduction efficiency observed with AAV based vectors in male mice compared to female mice, numerous initial AAV-related gene therapy studies only used single sex mice thereby increasing the likelihood of observing a therapeutic benefit, with the simplicity and reduce cost of such studies [23, 25–32]. In addition, the effect of sexual maturity has been overlooked so far. Pre-clinical studies using both genders with AAV based vectors are therefore carried out later in the development pathway. Pre-clinical efficacy studies in mice using systemic delivery of LVV are not very common, and the relatively few reports published only use male mice or don’t describe the sex of the animals [2, 3, 6]. Identifying the differences in sex-dependent transduction efficiencies in gene therapy candidates is important because most studies do not factor these differences. A recent report using a therapeutic AAV8 product candidate for MPSIVA where correction was only observed in male mice highlights the importance of using both male and female mice in efficacy studies [12] in that both groups need to be evaluated to inform pre-clinical efficacy of product candidates. Therefore, accounting for sex-specific differences is a critical variable for the development of safe and efficacious AAV and LVV gene therapy candidates.

In summary, we report for the first-time sex-specific differences in LVV transduction efficiencies and transgene expression in pre-clinical models, similar to those reported for rAAV.

## Data Availability

The original contributions presented in the study are included in the article/supplementary material, further inquiries can be directed to the corresponding author.

## References

[1] Bulcha JT Wang Y Ma H Tai PWL Gao G. Viral vector platforms within the gene therapy landscape. Signal Transduction Targeted Ther (2021) 6:53. 10.1038/s41392-021-00487-6 PMC7868676 33558455

[2] Carbonaro DA Jin X Petersen D Wang X Dorey F Kil KS *In vivo* transduction by intravenous injection of a lentiviral vector expressing human ADA into neonatal ADA gene Knockout mice: a novel form of enzyme replacement therapy for ada deficiency. Mol Ther (2006) 13:1110–20. 10.1016/j.ymthe.2006.02.013 16651028

[3] Carbonaro-Sarracino DA Tarantal AF Lee CCI Kaufman ML Wandro S Jin X Dosing and Re-administration of lentiviral vector for *in vivo* gene therapy in rhesus monkeys and ADA-deficient mice. Mol Ther - Methods and Clin Development (2020) 16:78–93. 10.1016/j.omtm.2019.11.004 PMC6909201 31871959

[4] Dalsgaard T Cecchi CR Askou AL Bak RO Andersen PO Hougaard D Improved lentiviral gene delivery to mouse liver by hydrodynamic vector injection through tail vein. Mol Ther - Nucleic Acids (2018) 12:672–83. 10.1016/j.omtn.2018.07.005 30092403 PMC6083003

[5] Brown BD Cantore A Annoni A Sergi LS Lombardo A Della Valle P A microRNA-regulated lentiviral vector mediates stable correction of hemophilia B mice. Blood (2007) 110:4144–52. 10.1182/blood-2007-03-078493 17726165

[6] Milani M Canepari C Liu T Biffi M Russo F Plati T Liver-directed lentiviral gene therapy corrects hemophilia A mice and achieves normal-range factor VIII activity in non-human primates. Nat Commun (2022) 13:2454. 10.1038/s41467-022-30102-3 35508619 PMC9068791

[7] Cantore A Ranzani M Bartholomae CC Volpin M Valle PD Sanvito F Liver-directed lentiviral gene therapy in a dog model of hemophilia B. Sci Transl Med (2015) 7:277ra28. 10.1126/scitranslmed.aaa1405 PMC5669486 25739762

[8] Nicolas CT VanLith CJ Hickey RD Du Z Hillin LG Guthman RM *In vivo* lentiviral vector gene therapy to cure hereditary tyrosinemia type 1 and prevent development of precancerous and cancerous lesions. Nat Commun (2022) 13:5012. 10.1038/s41467-022-32576-7 36008405 PMC9411607

[9] Berraondo P Crettaz J Ochoa L Pañeda A Prieto J Trocóniz IF Intrahepatic injection of recombinant adeno-associated virus serotype 2 overcomes gender-related differences in liver transduction. Hum Gene Ther (2006) 17:601–10. 10.1089/hum.2006.17.601 16776569

[10] Davidoff AM Ng CYC Zhou J Spence Y Nathwani AC. Sex significantly influences transduction of murine liver by recombinant adeno-associated viral vectors through an androgen-dependent pathway. Blood (2003) 102:480–8. 10.1182/blood-2002-09-2889 12637328

[11] Mochizuki S Mizukami H Ogura T Kure S Ichinohe A Kojima K Long-term correction of hyperphenylalaninemia by AAV-mediated gene transfer leads to behavioral recovery in phenylketonuria mice. Gene Ther (2004) 11:1081–6. 10.1038/sj.gt.3302262 15057263

[12] Piechnik M Amendum PC Sawamoto K Stapleton M Khan S Fnu N Sex difference leads to differential gene expression patterns and therapeutic efficacy in mucopolysaccharidosis IVA murine model receiving AAV8 gene therapy. Int J Mol Sci (2022) 23:12693. 10.3390/IJMS232012693 36293546 PMC9604118

[13] Vigna E Amendola M Benedicenti F Simmons AD Follenzi A Naldini L. Efficient tet-dependent expression of human factor IX *in vivo* by a new self-regulating lentiviral vector. Mol Ther (2005) 11:763–75. 10.1016/j.ymthe.2004.11.017 15851015

[14] Iqball S Beck DK Devarajan G Khoo CP O’Connor DM Ellis S Lentiviral delivered aflibercept OXB-203 for treatment of neovascular AMD. Mol Ther - Methods and Clin Development (2023) 30:350–66. 10.1016/j.omtm.2023.07.001 PMC10448334 37637380

[15] Senova S Poupon C Dauguet J Stewart HJ Dugué GP Jan C Optogenetic Tractography for anatomo-functional characterization of cortico-subcortical neural circuits in non-human primates. Sci Rep (2018) 8:3362. 10.1038/s41598-018-21486-8 29463867 PMC5820256

[16] Pañeda A Vanrell L Mauleon I Crettaz JS Berraondo P Timmermans EJ Effect of adeno-associated virus serotype and genomic structure on liver transduction and biodistribution in mice of both genders. Hum Gene Ther (2009) 20:908–17. 10.1089/hum.2009.031 19419275

[17] Shedlovsky A McDonald JD Symula D Dove WF. Mouse models of human phenylketonuria. Genetics (1993) 134:1205–10. 10.1093/genetics/134.4.1205 8375656 PMC1205587

[18] Balaggan KS Binley K Esapa M Iqball S Askham Z Kan O Stable and efficient intraocular gene transfer using pseudotyped EIAV lentiviral vectors. The J Gene Med (2006) 8:275–85. 10.1002/jgm.845 16299834

[19] Campochiaro PA Lauer AK Sohn EH Mir TA Naylor S Anderton MC Lentiviral vector gene transfer of endostatin/angiostatin for macular degeneration (GEM) study. Hum Gene Ther (2017) 28:99–111. 10.1089/hum.2016.117 27710144 PMC5278797

[20] Bell P Gao G Haskins ME Wang L Sleeper M Wang H Evaluation of adeno-associated viral vectors for liver-directed gene transfer in dogs. Hum Gene Ther (2011) 22:985–97. 10.1089/hum.2010.194 21204705 PMC3159528

[21] Arruda V Scallan C Jian H Couto L Herzog R Nichols T Comparison of the efficacy on gene transfer by AAV vectors delivered by distinct routes of administration to the liver of hemophilia B dogs. Mol Ther (2004) 9:S40–S41.

[22] Kaiser RA Weber ND Trigueros‐Motos L Allen KL Martinez M Cao W Use of an adeno‐associated virus serotype Anc80 to provide durable cure of phenylketonuria in a mouse model. J Inherit Metab Dis (2021) 44:1369–81. 10.1002/jimd.12392 33896013 PMC9291745

[23] Han SO Gheorghiu D Chang A Mapatano SH Li S Brooks E Efficacious androgen hormone administration in combination with adeno-associated virus vector-mediated gene therapy in female mice with pompe disease. Hum Gene Ther (2022) 33:479–91. 10.1089/hum.2021.218 35081735 PMC9142766

[24] Han S Li S McCall A Arnson B Everitt JI Zhang H Comparisons of infant and adult mice reveal age effects for liver depot gene therapy in pompe disease. Mol Ther - Methods and Clin Development (2020) 17:133–42. 10.1016/j.omtm.2019.11.020 PMC6938806 31909086

[25] Maturana CJ Chan A Verpeut JL Engel EA. Local and systemic administration of AAV vectors with alphaherpesvirus latency-associated promoter 2 drives potent transgene expression in mouse liver, kidney, and skeletal muscle. J Virol Methods (2023) 314:114688. 10.1016/j.jviromet.2023.114688 36736702 PMC10236909

[26] Bell P Wang L Chen S-J Yu H Zhu Y Nayal M Effects of self-complementarity, codon optimization, transgene, and dose on liver transduction with AAV8. Hum Gene Ther Methods (2016) 27:228–37. 10.1089/hgtb.2016.039 27903094 PMC6445178

[27] Wang L Wang H Morizono H Bell P Jones D Lin J Sustained correction of OTC deficiency in spfash mice using optimized self-complementary AAV2/8 vectors. Gene Ther (2012) 19:404–10. 10.1038/gt.2011.111 21850052 PMC3321078

[28] Cunningham SC Spinoulas A Carpenter KH Wilcken B Kuchel PW Alexander IE. AAV2/8-mediated correction of OTC deficiency is robust in adult but not neonatal spfash mice. Mol Ther (2009) 17:1340–6. 10.1038/mt.2009.88 19384294 PMC2835243

[29] Bell P Wang L Lebherz C Flieder DB Bove MS Wu D No evidence for tumorigenesis of AAV vectors in a large-scale study in mice. Mol Ther (2005) 12:299–306. 10.1016/j.ymthe.2005.03.020 16043099

[30] Kok CY Tsurusaki S Cabanes-Creus M Igoor S Rao R Skelton R Development of new adeno-associated virus capsid variants for targeted gene delivery to human cardiomyocytes. Mol Ther - Methods and Clin Development (2023) 30:459–73. 10.1016/j.omtm.2023.08.010 PMC10477751 37674904

[31] Anderson JM Arnold WD Huang W Ray A Owendoff G Cao L. Long-term effects of a fat-directed FGF21 gene therapy in aged female mice. Gene Ther (2023) 31:95–104. 10.1038/s41434-023-00422-0 37699965 PMC12997126

[32] Sun B Zhang H Franco LM Young SP Schneider A Bird A Efficacy of an adeno-associated virus 8-pseudotyped vector in glycogen storage disease type II. Mol Ther (2005) 11:57–65. 10.1016/j.ymthe.2004.10.004 15585406

